# Identification and Verification of Core Genes in Colorectal Cancer

**DOI:** 10.1155/2020/8082697

**Published:** 2020-05-08

**Authors:** Houxi Xu, Yuzhu Ma, Jinzhi Zhang, Jialin Gu, Xinyue Jing, Shengfeng Lu, Shuping Fu, Jiege Huo

**Affiliations:** ^1^Key Laboratory of Acupuncture and Medicine Research of the Ministry of Education, Nanjing University of Chinese Medicine, Nanjing 210046, China; ^2^Affiliated Hospital of Integrated Traditional Chinese and Western Medicine, Nanjing University of Chinese Medicine, Nanjing 210028, China

## Abstract

Colorectal cancer, a malignant neoplasm that occurs in the colorectal mucosa, is one of the most common types of gastrointestinal cancer. Colorectal cancer has been studied extensively, but the molecular mechanisms of this malignancy have not been characterized. This study identified and verified core genes associated with colorectal cancer using integrated bioinformatics analysis. Three gene expression profiles (GSE15781, GSE110223, and GSE110224) were downloaded from the Gene Expression Omnibus (GEO) databases. A total of 87 common differentially expressed genes (DEGs) among GSE15781, GSE110223, and GSE110224 were identified, including 19 upregulated genes and 68 downregulated genes. Gene ontology and Kyoto Encyclopedia of Genes and Genomes pathway enrichment analysis was performed for common DEGs using clusterProfiler. These common DEGs were significantly involved in cancer-associated functions and signaling pathways. Then, we constructed protein-protein interaction networks of these common DEGs using Cytoscape software, which resulted in the identification of the following 10 core genes: SST, PYY, CXCL1, CXCL8, CXCL3, ZG16, AQP8, CLCA4, MS4A12, and GUCA2A. Analysis using qRT-PCR has shown that SST, CXCL8, and MS4A12 were significant differentially expressed between colorectal cancer tissues and normal colorectal tissues (*P* < 0.05). Gene Expression Profiling Interactive Analysis (GEPIA) overall survival (OS) has shown that low expressions of AQP8, ZG16, CXCL3, and CXCL8 may predict poor survival outcome in colorectal cancer. In conclusion, the core genes identified in this study contributed to the understanding of the molecular mechanisms involved in colorectal cancer development and may be targets for early diagnosis, prevention, and treatment of colorectal cancer.

## 1. Introduction

Colorectal cancer is the third most prevalent cancer worldwide and has the second highest mortality rate among all cancers [1]. The global burden of colorectal cancer is expected to increase by 60% by 2030, with >2.2 million new cases and 1.1 million deaths [2]. Early symptoms of colorectal cancer are atypical and are often missed or misdiagnosed. Colorectal cancer is typically detected at middle or late stages of the disease. Commonly used early detection methods for colorectal cancer include fecal-based examination, blood-based examination, and enteroscopy [3]. However, several instrument-dependent detection methods may not be available in regions with limited economic resources. The main treatment options for colorectal cancer are surgery, neoadjuvant radiotherapy (for rectal cancer), adjuvant chemotherapy (for stage III/IV and high-risk stage II colon cancer), and molecular-targeted drug therapy [4, 5]. However, these treatments have some drawbacks. Studies have shown that less than 15% of metastatic colorectal cancer is suitable for surgical resection, the recurrence rate exceeds 80% within 3 years after surgery, and the recurrence rate exceeds 95% within 5 years after surgery [6]. In addition, tumor cells have innate tolerance or develop tolerance, to radiotherapy and chemotherapy [7]. Although molecular-targeted drugs can extend the median survival time of patients with colon cancer, the 5-year survival rate of patients is relatively low [5]. Advances in treatment of colorectal cancer have been made, but the 5-year survival rate of patients with this disease has not improved significantly. Therefore, identifying new biomarkers to understand the molecular changes that drive colorectal cancer is critical to achieving effective strategies for early diagnosis and new target therapies.

Genes control cell proliferation, apoptosis, differentiation, migration, and invasion, and imbalances in gene expression occur in a variety of human cancers. Recent studies have evaluated gene expression in colorectal cancer using microarray dataset or next-generation sequencing technology. The new technologies can help identify core genes related to the development of colorectal cancer, and these core genes are considered to be effective candidates for biomarkers in the development of colorectal cancer. Therefore, screening of core genes for colorectal cancer is very important. For example, Sole et al. used a microarray dataset to identify 505 unique candidate biomarkers of colorectal cancer [8]. Zhao et al. identified 131 differentially expressed genes (DEGs) in 12 patients with colorectal cancer and 10 healthy controls, among which 108 were upregulated and 23 were downregulated [9]. Chen et al. identified 428 upregulated genes and 751 downregulated genes in colorectal cancer. The functional changes associated with these DEGs were mainly related to cell cycle, oocyte meiosis, DNA replication, p53 signaling pathway, and progesterone-mediated oocyte maturation [10]. However, these studies suffered from some limitations. (1) First, most colorectal cancer studies have used a single GEO dataset for core gene analysis [8–10]. Due to differences in microarray platforms and sample specificity, core genes analyzed using a single GEO dataset may not be accurate. The three examples mentioned above all used a single microarray to study DEGs in colorectal cancer. (2) Secondly, few studies have used multiple microarray datasets to study core genes in colorectal cancer. Guo et al. identified 292 common DEGs in four microarrays, of which 165 were upregulated and 127 were downregulated, and 31 core genes were identified using Cytoscape software [11]. However, the study by Guo et al. did not collect clinical colorectal cancer samples to verify the identified core genes. These limitations highlight the need to use diverse datasets and to verify core genes identified during analysis of these datasets. Therefore, in order to discover potential biomarkers of colorectal cancer and solve the above problems, we conducted this study.

In this study, we used three Gene Expression Omnibus (GEO) datasets to identify core genes associated with colorectal cancer and used quantitative real-time PCR (qRT-PCR) and GEPIA website to verify the expression of core genes in clinical colorectal cancer samples. Gene expression data from GSE15781 [12], GSE110223 [13], and GSE110224 [13] were downloaded from the GEO database. These datasets included 43 colorectal cancer tissue data and 40 colorectal normal tissue data. Eighty-seven common DEGs were identified using the intersection function of R software, of which 19 were upregulated and 68 were downregulated. Gene ontology (GO) and Kyoto Gene and Genomic Encyclopedia (KEGG) pathway enrichment analysis was performed on the common DEGs using the clusterProfiler package in R software. The common DEGs were significantly enriched in multiple cancer-related functions and pathways. The STRING online database was used to construct a protein-protein interaction (PPI) network of common DEGs, and the core genes in the PPI network was identified using the MCODE plug-in of Cytoscape software. A total of 10 core genes associated with colorectal cancer were identified. Finally, the core genes were verified using qRT-PCR and GEPIA. Quantitative RT-PCR results have shown that the following 3 core genes were significantly different between colorectal cancer tissues and normal colorectal tissues: SST, CXCL8, and MS4A12. Gene Expression Profiling Interactive Analysis (GEPIA) overall survival (OS) analysis has shown that low expressions of AQP8, ZG16, CXCL3, and CXCL8 may predict poor survival outcome in colorectal cancer. In conclusion, this study will provide potential biomarkers for the diagnosis, prognosis, and new therapeutic targets of colorectal cancer.

## 2. Materials and Methods

### 2.1. Gene Microarray Data Download

The keyword “colorectal cancer microarray human” was searched in the GEO database, and three GEO datasets (GSE15781 [12], GSE110223 [13], and GSE110224 [13]) were randomly selected for download. Data from 83 samples (43 colorectal cancer tissue samples and 40 normal colorectal tissue samples) were included. In the GSE15781 dataset, published in February 2010 and based on the GPL2986 platform (ABI Human Genome Survey Microarray Version 2), the data taken from tissue samples of colorectal cancer patients includes 13 cancer tissue and 10 adjacent tissue. In the GSE110223 dataset, published in January 2019 and based on the GPL96 platform (HG-U133A, Affymetrix Human Genome U133A Array), the data taken from tissue samples of colorectal cancer patients includes 13 cancer tissue and 13 adjacent tissue. In the GSE110224 dataset, published in January 2019 and based on the GPL570 platform (HG-U133_Plus_2, Affymetrix Human Genome U133 Plus 2.0 Array), the data taken from tissue samples of colorectal cancer patients includes 17 cancer tissue and 17 adjacent tissue. Convert the probe ID in the download file to the gene SYMBOL name by using R software. All gene expression data were standardized and log2 transformed.

### 2.2. Identification of Common DEGs in Colorectal Cancer

Analysis of DEGs was performed using the limma package of R software [14]. The identification criteria for DEGs were as follows: *P* value < 0.05 and log∣FC∣ > 1. The intersection function of R software was used to identify common DEGs among the three GEO datasets. A Venn diagram to represent the DEGs among the datasets was generated using the VennDiagram R package.

### 2.3. GO and KEGG Pathway Enrichment Analyses of Common DEGs

clusterProfiler is a tool based on GO that provides groupGO, enrichGO, and enrichKEGG for genetic classification and enrichment analysis [15]. clusterProfiler is easy to use, and it provides a visual output of the analysis results. To explore the biological functions of common DEGs, GO and KEGG pathway enrichment analyses were performed on the common DEGs using the clusterProfiler package. *P* value < 0.05 was considered statistically significant.

### 2.4. Construction of a PPI Network

The STRING website (http://string-db.org/) is a database for identifying interactions between known and predicted proteins and is often used to construct PPI networks [16]. We used the STRING website to build a PPI network for common DEGs. Cytoscape software (http://www.cytoscape.org/) is an open-source web visualization software platform based on Java technology [17]. It is commonly used to visualize PPI networks, miRNA-gene networks, and ceRNA networks. To visualize and analyze PPI networks of common DEGs, the data were imported into Cytoscape software and processed using the default parameters of the software.

### 2.5. Identification of Core Genes Associated with Colorectal Cancer

MCODE is a plug-in for Cytoscape software used to mine functional modules in biological networks [18]. The genes involved in the functional modules are considered to be core genes. We used the MCODE plug-in to analyze the PPI network of common DEGs for functional modules and core genes. The parameter settings of the MCODE plug-in were as follows: degree = 5, node score = 0.2, *k* − core = 5, max.depth = 100.

### 2.6. Verification of Core Genes

Total RNA was extracted from tissues using TRIzol reagent. Total RNA was reverse transcribed into cDNA using the PrimeScript RT Reagent Kit (Takara, Dalian, China). Primers were designed using Primer BLAST. The expression levels of genes were measured using a qRT-PCR system (Applied Biosystems, Foster City, CA, USA). GAPDH was used as the internal reference gene. The data were analyzed using the comparative cycle threshold (CT, 2^-*Δ*ΔCT^) method. A total of 16 pairs of specimens (colorectal cancer tissues and adjacent normal samples) were obtained from colorectal cancer patients who received surgical treatment between October 2019 and December 2019 at Jiangsu Provincial Hospital of Integrated Traditional Chinese and Western Medicine. This study was approved by the Ethics Committee of the Jiangsu Provincial Hospital of Integrated Traditional Chinese and Western Medicine (2019LWKYZ007). All patients provided written informed consent prior to collection of specimens. qRT-PCR program settings are as follows: predenaturation at 95°C for 5 min, denaturation at 95°C for 10 sec, annealing/extension at 60°C for 30 sec, and repeat 40 cycles; other parameters are performed according to the instrument default settings. For detailed information regarding qRT-PCR primer of genes, please refer to Supplementary Material Table S1.

GEPIA (http://gepia.cancer-pku.cn/index.html) is a tool website that analyzes The Cancer Genome Atlas (TCGA) data and determine the OS outcomes [19].

## 3. Results

### 3.1. Identification of Common DEGs in Colorectal Cancer

**Figure 1:**
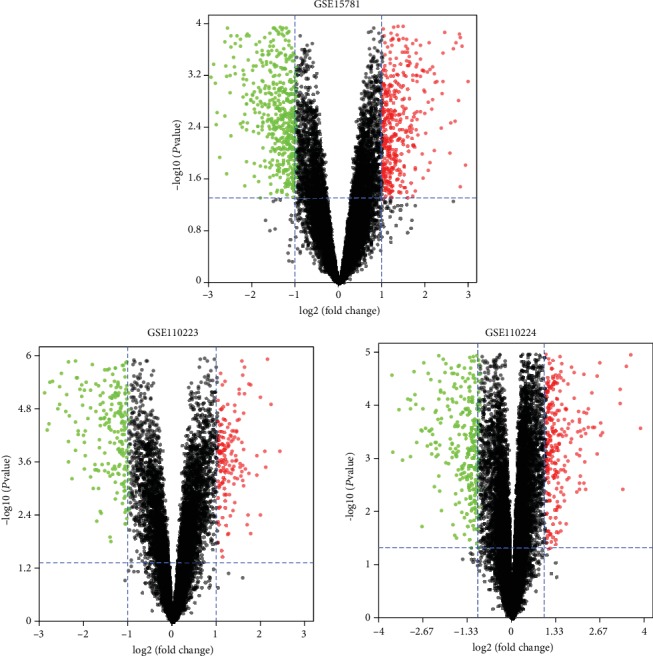
Volcano plots of the three GEO datasets. Red represents upregulated genes, and green represents downregulated genes (*P* < 0.05 and log∣FC∣ > 1).

**Figure 2:**
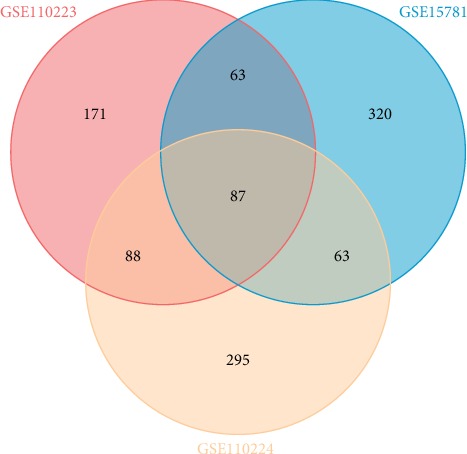
Common DEGs among the three GEO datasets.

The gene expression data were processed and normalized, and DEGs among each GEO datasets were identified using the limma package with a *P* value < 0.05 and log∣FC∣ > 1 set as thresholds. From the GSE15781 dataset, 533 DEGs were screened, including 204 upregulated genes and 329 downregulated genes. From the GSE110223 dataset, 408 DEGs were screened, including 154 upregulated genes and 254 downregulated genes. From the GSE110224 dataset, 532 DEGs were screened, including 241 upregulated genes and 291 downregulated genes. Volcano plots of the three GEO datasets are shown in Figure 1. The intersection function of R software was used to identify common DEGs among the three GEO datasets. As shown in Figure 2, 87 common DEGs were identified among the three GEO datasets, including 19 upregulated genes and 68 downregulated genes. We have listed all of the upregulated common DEGs and downregulated common DEGs in Table 1.

### 3.2. GO and KEGG Pathway Enrichment Analyses of Common DEGs

**Figure 3:**
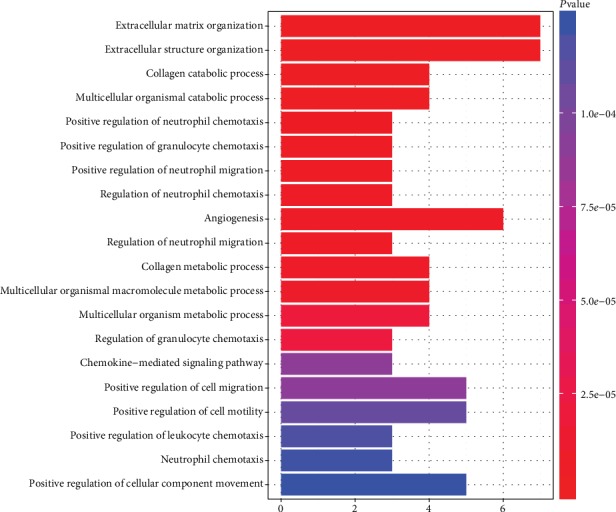
The results for GO analysis of upregulated common DEGs.

**Figure 4:**
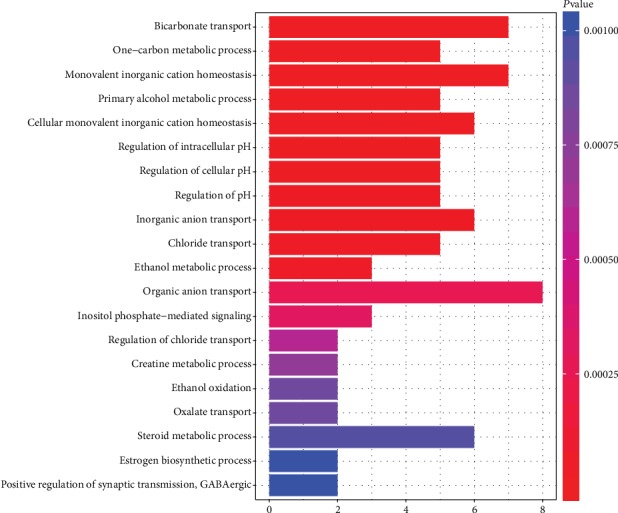
The results for GO analysis of downregulated common DEGs.

GO analysis was performed on the upregulated and downregulated common DEGs using the clusterProfiler package. The screening criterion for GO analysis was a *P* value < 0.05. Gene ontology analysis results for the common DEGs included molecular function, biological process, and cellular components. In biological processes, the upregulated genes were mainly involved in positive regulation of neutrophil migration, organization of the extracellular matrix, and collagen catabolic process. The downregulated genes were mainly enriched in bicarbonate transport, regulation of cellular pH, and homeostasis of monovalent inorganic cations. In cellular components, the upregulated genes were enriched in proteinaceous extracellular matrix, basement membrane, and extracellular matrix. The downregulated genes were mainly enriched in the apical part of a cell, apical plasma membrane, and actin-based cell projection. In molecular functions, the upregulated genes were enriched in CXCR chemokine receptor binding, chemokine activity, and G-protein-coupled receptor binding. The downregulated genes were mainly enriched in oxidoreductase activity, steroid dehydrogenase activity, and bicarbonate transmembrane transporter activity. The results for GO analysis of upregulated common DEGs and downregulated common DEGs are shown in Figures 3 and 4. The detailed results of GO analysis in Supplementary Materials Tables S2 and S3, respectively.

**Figure 5:**
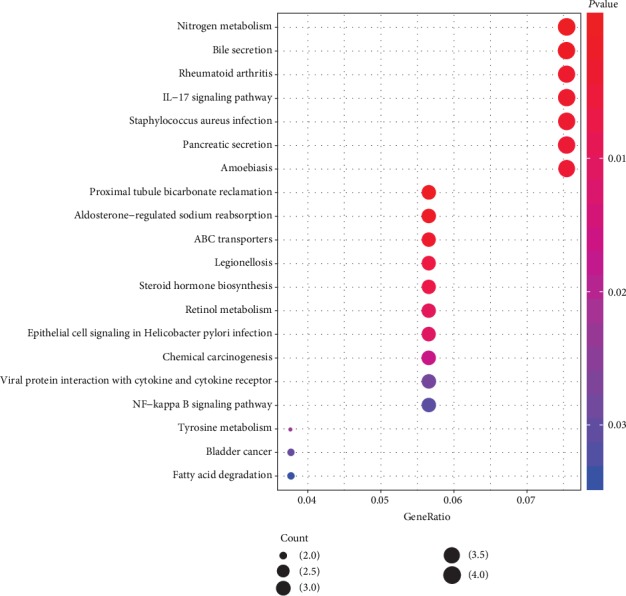
KEGG pathway enrichment analysis of common DEGs.

Kyoto Encyclopedia of Genes and Genomes pathway enrichment analysis was performed on the common DEGs using the clusterProfiler package. The screening criterion for KEGG pathway enrichment analysis was a *P* value < 0.05. The KEGG pathway enrichment analysis results for common DEGs are shown in Figure 5. Eighteen signaling pathways were identified. The signaling pathways of common DEGs were mainly enriched in the cytokine-cytokine receptor interaction, IL-17, and ABC transporter signaling pathways. These results have shown that the common DEGs were significantly enriched in cancer-related biological processes.

### 3.3. Construction of a PPI Network and Identification of Core Genes

**Figure 6:**
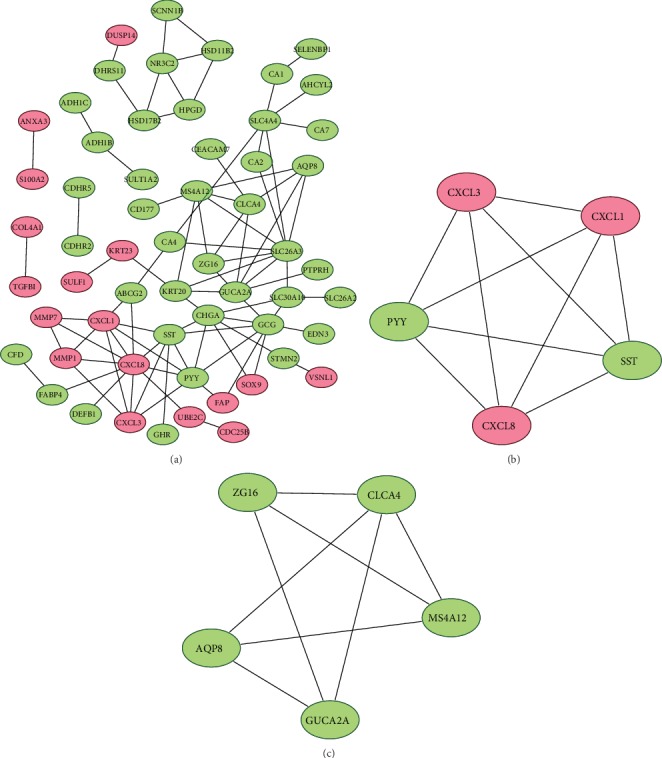
PPI network and subnetwork: (a) PPI network of common DEGs, (b) functional module 1 of PPI network, and (c) functional module 2 of PPI network.

To further evaluate the interactions between the identified common DEGs, we used the STRING website to construct a PPI network. The PPI network of common DEGs is shown in Figure 6(a), and it consists of 86 edges and 59 nodes, including 17 upregulated genes and 42 downregulated genes. We found two functional modules from the PPI network using the MCODE plug-in (Figures 6(b) and 6(c)). Functional module 1 contained 5 genes: PYY, SST, CXCL3, CXCL1, and CXCL8. Functional module 2 contained 5 genes: ZG16, AQP8, CLCA4, MS4A12, and GUCA2A. The 10 genes in these two functional modules were considered to be the core genes associated with colorectal cancer.

### 3.4. Verification of Core Genes

**Figure 7:**
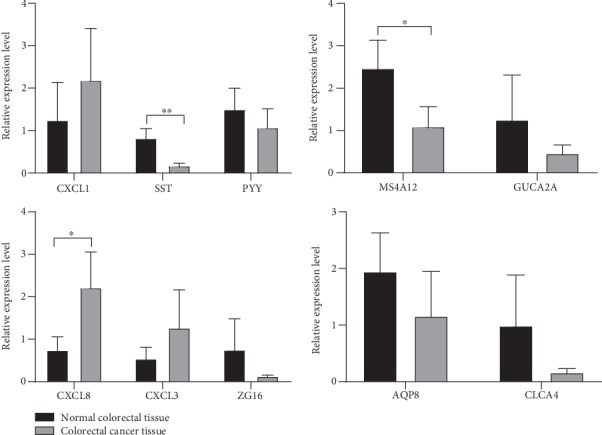
Quantitative RT-PCR verification of core genes.

To verify the authenticity of the 10 core genes, we obtained colorectal cancer tissues and normal colorectal tissues from 16 patients with colorectal cancer for analysis using qRT-PCR. The qRT-PCR results shown in Figure 7 demonstrated that SST, CXCL8, and MS4A12 were significantly differentially expressed between colorectal cancer tissue and normal colorectal tissue (*P* < 0.05). In contrast, PYY, CXCL3, GUCA2A, CXCL1, ZG16, CXCL3, and AQP8 were not differentially expressed.

**Figure 8:**
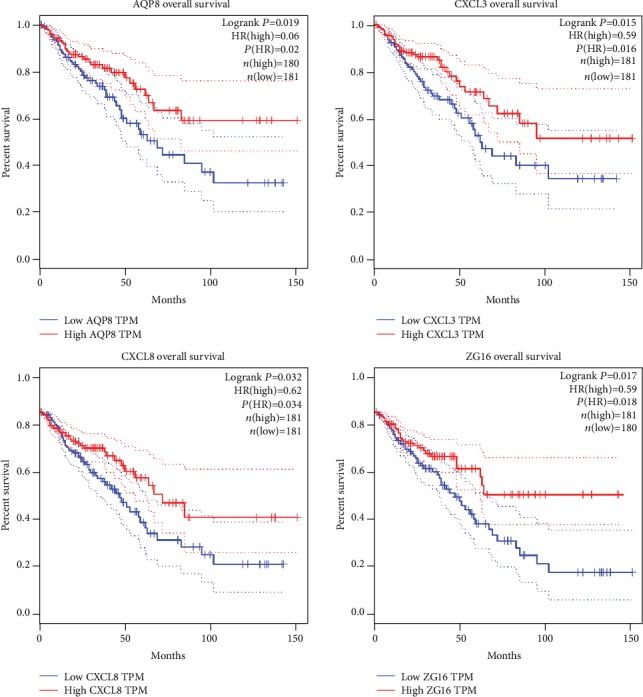
Overall survival verification of core genes.

The OS analysis of 10 core genes was performed by using the GEPIA tool website, and the results show that low expressions of AQP8, ZG16, CXCL3, and CXCL8 may predict poor survival outcome in colorectal cancer. The OS analysis results are shown in Figure 8.

## 4. Discussion

Colorectal cancer is one of the most common malignant tumors of the digestive system. According to statistical surveys, colorectal cancer has the third highest incidence among the common cancers worldwide, with more than 1.4 million new cases each year [20, 21]. The global burden of colorectal cancer is expected to increase by 60% by 2030, with >2.2 million new cases and 1.1 million deaths [2]. Early symptoms of colorectal cancer are atypical and are easily missed or misdiagnosed. The main treatment strategies for colorectal cancer are surgery, neoadjuvant radiotherapy, adjuvant chemotherapy, and molecular-targeted drug therapy [4, 5]. Despite the existence of many treatments for colorectal cancer, the 5-year survival rate for colorectal cancer remains below 40% due to recurrence and metastasis [22, 23]. Therefore, identifying new biomarkers to understand the molecular changes that drive colorectal cancer is critical to achieving effective strategies for early diagnosis and new target therapies.

Microarray technology and next-generation sequencing technology have been widely used in cancer research, including colorectal cancer research. The use of new technologies can help identify core genes related to the development of colorectal cancer. These core genes are considered to be effective candidates for biomarkers in the development of colorectal cancer. Therefore, the core genes of colorectal cancer can be screened before screening for biomarkers of colorectal cancer. Based on these techniques, aberrantly significant genes and pathways have been found in colorectal cancer. However, these studies suffered from some limitations, including the following: (1) First, most colorectal cancer studies have used a single GEO dataset for core gene analysis [8–10]. Due to differences in microarray platforms and sample specificity, core genes analyzed using a single GEO dataset may not be accurate. (2) Secondly, a small number of colorectal cancer studies have used multiple GEO datasets, but these studies did not verify the identified core genes [11]. These limitations highlight the need to use diverse datasets and to verify core genes identified during analysis of these datasets.

In this study, we identified and verified core genes associated with colorectal cancer using bioinformatics methods. We identified 87 common DEGs among three GEO datasets, including 19 upregulated genes and 68 downregulated genes. GO analysis of the common DEGs has shown that the upregulated common DEGs were mainly involved in neutrophil activity, receptor regulation, chemokine activity, and extracellular matrix. This finding was consistent with the concept that tumors control neutrophil activity by presenting various phenotypic and functional polarization states to alter tumor behavior. The downregulated common DEGs were mainly enriched in bicarbonate transport, regulation of cellular pH, steroid dehydrogenase activity, and apical part of cell. This finding is in line with a study that states bicarbonate transport plays an important role in the diagnosis and treatment of many cancers [24]. KEGG pathway enrichment analysis has shown that the signaling pathways of the common DEGs were mainly enriched in cytokine-receptor interaction, IL-17, and ABC transporter signaling pathways. Interleukin 17 is an inflammatory cytokine upregulated in the serum and tissues of patients with colorectal cancer. It is strongly associated with onset, angiogenesis, metastasis, diagnosis, prognosis, and treatment of colorectal cancer [25]. In addition, epidermal growth factor receptor (EGFR) has been shown to be involved in pathogenesis and progression of cancer, and the cytokine-receptor interaction signaling pathway is an important pathway in the development of cancer [26]. The ABC super family of transporters are encoded by 49 genes contained in the human genome. Eight ABC transporter subfamilies have been identified based on transporter homology and structural similarity [27]. A study suggested that downregulation of ABC transporters in nonneoplastic tissues may be associated with better prognoses for pancreatic cancer and colorectal cancer [28]. The common DEGs identified in our study were shown to be significantly involved in cancer-related functions and signaling pathways.

Ten core genes (SST, PYY, CXCL1, CXCL8, CXCL3, ZG16, AQP8, CLCA4, MS4A12, and GUCA2A) with the most important interactions were selected from the PPI network. In addition, 3 core genes (SST, CXCL8, and MS4A12) were found to be significantly differentially expressed between colorectal cancer tissue and normal colorectal tissue using qRT-PCR. In contrast, PYY, CXCL3, GUCA2A, ZG16, CLCA4, CXCL1, and AQP8 were not differentially expressed using qRT-PCR. The results of OS analysis have shown that low expressions of AQP8, ZG16, CXCL3, and CXCL8 may predict poor survival outcome in colorectal cancer. Therefore, CXCL8 is more competitive as a candidate biomarker than other core genes.

Somatostatin (SST) is a polypeptide hormone that is widely distributed in the human body and exerts antiproliferative and proapoptotic effects. The inhibitory effects of SST on tumor cells have received increasing attention in recent years. A large number of in vitro and in vivo experiments have proved that SST and somatostatin analogue (SSTA) recognized the somatostatin receptor (SSTR) on the cell membrane and specifically bind to it to generate a transmembrane signal, thereby exerting biological effects and directly or indirectly inhibiting tumor growth [29, 30]. Therefore, SST and SSTA are likely to develop into a new class of antitumor drugs. Shields et al. found that SSTA recognizes SSTR2 and combines with it to activate tyrosine phosphatase SHP-1, thereby terminating the effects of growth factors and cytokines through dephosphorylation and directly inhibiting tumor cell proliferation [29]. Schoppmann et al. found that SSTA recognizes SSTR-3 and combines with it to activate endonucleases, thereby enhancing bax expression and finally inducing apoptosis [30]. In few studies on the role of SST in colorectal cancer, we speculate that SST has a similar role in colorectal cancer.

Interleukin 8 (CXCL8), CXCL3, and CXCL1 are CXC chemokines that are strongly associated with tumor angiogenesis. According to glutamate leucine arginine (ELR) function, CXC chemokines are classified into ELR chemokines and non-ELR chemokines. ELR chemokines induce tumor angiogenesis, and non-ELR chemokines inhibit tumor angiogenesis. ELR chemokines include CXCL8, CXCL3, and CXCL1, which promote tumor angiogenesis [31]. Ning et al. found that whether in vivo or in vitro, overexpression of CXCL8 promotes tumor growth, metastasis, and angiogenesis, which means that CXCL8 may be an important therapeutic target for colorectal cancer [32]. Wang et al. used prostaglandin E2 to induce CXCL1 overexpression to promote angiogenesis in colorectal cancer [33]. Chen et al. have demonstrated that CXCL5 induces tumor angiogenesis via enhancing the expression of FOXD1 mediated by the AKT/NF-*κ*B pathway in colorectal cancer [34]. In addition, our study also found that CXCL8, CXCL3, and CXCL1 are involved in the IL-17 signaling pathway. Studies have shown that IL-17 induces granulocyte formation through production of granulocyte colony-stimulating factors and induces the expression of CXC chemokines [35]. Therefore, the CXC family plays an important role in the development of colorectal cancer, especially CXCL8, CXCL3, and CXCL1.

Zymogen granule protein 16 (ZG16) is a soluble protein that is expressed only in epithelial cells of the small intestine, rectum, and colon [36]. Studies have shown that ZG16 is downregulated in colorectal cancer, which is consistent with the results of our analysis [37–39]. However, the detailed molecular mechanism of ZG16 in colorectal cancer is still unknown. Meng et al. speculated that the loss of ZG16 may promote invasion of bacteria to the host system and cause local inflammation, increasing the risk of cancer development [38].

Calcium-activated chloride channel 4 (CLCA4) is a member of the calcium-activated chloride channel protein family and is expressed in intestinal epithelial cells and in breast, uterus, prostate, epididymis, testis, and brain tissue [40]. CLCA4 is also known as a tumor suppressor, which can promote the development of many types of malignant tumors. Studies have shown that CLCA4 can inhibit tumor differentiation in breast cancer. After knockout of CLCA4, it is found that tumors induce tumor cell differentiation and metastasis through epithelial-mesenchymal transition [41]. The in vitro experiments in colorectal cancer show that overexpression of CLCA4 can inhibit cancer cell migration and invasion by suppressing epithelial-mesenchymal transition (EMT) via PI3K/ATK signaling [42].

Peptide tyrosine-tyrosine (PYY), as a gastrointestinal hormone, has a number of important regulatory effects on the physiological functions of the digestive tract, including affecting gastrointestinal movement and inhibiting intestinal mucosal secretion. Recent studies have shown that PYY exists in a variety of tumor tissues, and the decrease in its expression may be related to the occurrence and progression of tumors, and it has an inhibitory function on a variety of tumors [43–46]. This is also consistent with the results of our analysis. However, the detailed mechanism by which PYY inhibits tumor cells is unclear. Kling et al. found that PYY can combine with the PYY receptor, thereby inhibiting the growth of human colorectal cancer Caco-2 and HT-116 cell lines [47]. This may be one of the molecular mechanisms by which PYY inhibits cancer cells in colorectal cancer.

The aquaporins (AQPs) are composed of a series of small membrane transporters. They can be divided into two categories based on their permeability: AQP1, 2, 4, 5, and 8 are used as water-selective transporters; AQP3, 7, 9, and 10 can transport water, glycerol, and other small solutes [48]. Studies have shown that abnormal expression of AQP family members is related to tumorigenesis. For example, AQP5 is overexpressed in ovarian cancer, cervical cancer, and breast cancer [49–51]. Wu et al. found that AQP8 plays an important role in the growth and metastasis of colorectal cancer cells; the overexpression of AQP8 inhibited the growth and invasion of colorectal SW480 and HT-29 cells [52]. Mechanistically, the overexpression of AQP8 inhibits the expression of PCDH7 through the PI3K/AKT signaling pathway, thereby inhibiting the growth and metastasis of colorectal cancer cells. Therefore, the research of APQ8 for colorectal cancer is very important, and it is a potential therapeutic target for colorectal cancer patients.

Membrane spanning 4 domain subfamily A 12 (MS4A12), a member of the MS4A family, is specifically expressed in colonic epithelium. Members of the MS4A family play important roles in cell differentiation, signal transduction, and cell cycle regulation [53]. Koslowski et al. found that MS4A12 is regulated by intestinal tumor suppressor gene CDX2 in colon cancer and can affect colon cancer cell proliferation and cycle [54, 55]. The colon-specific expression of MS4A12 makes it a potential target for colon cancer immunotherapy. However, no research has explored the relationship between MS4A12 and the occurrence of colorectal cancer.

There are few studies on guanylate cyclase-activating factor 2A (GUCA2A), and the mechanisms are still unclear. GUCA2A combines with guanylate cyclase-activating factor 2B (GUCA2B) and activates guanylyl cyclase C (GUCY2C), thereby regulating intestinal proliferation, metabolism, and barrier functions. GUCY2C is a transmembrane receptor expressed on intestinal epithelial cells and plays an important role in coordinating the mechanism of intestinal homeostasis. Recent studies have shown that there is a link between GUCY2C silence and intestinal dysfunction, including tumorigenesis [56]. Therefore, we speculated that GUCA2A affects the development of colorectal cancer by regulating GUCY2C.

Compared with previous studies on colorectal cancer, this study has the following innovations: (1) integration of three microarray datasets instead of single microarrays; (2) bioinformatics-based GO and KEGG pathway enrichment analysis; and (3) identification of core genes using the MCODE plug-in and verification of core genes using qRT-PCR and GEPIA. These methods may be critical for identification of reliable biomarkers for colorectal cancer diagnosis and prognosis. Of course, our study also suffered from some limitations: (1) In this study, only 16 clinical samples were collected, which limited verification of core genes; and (2) we did not use western blot to verify the protein expression of core genes, which effected the authenticity of core genes. In future studies, we plan to collect a greater number of clinical samples to study the pathogenesis of colorectal cancer and use western blot to verify the protein expression of core genes.

## 5. Conclusions

We identified core genes related to colorectal cancer from the three GEO datasets by an integrated bioinformatics analysis, and 10 core genes were verified by using qRT-PCR and GEPIA. Our findings increased our understanding of the molecular mechanisms of colorectal cancer development and may contribute to the development of novel strategies for early diagnosis and prevention of colorectal cancer. Finally, our study may contribute to the identification of targets for the treatment of colorectal cancer.

## Figures and Tables

**Table 1:** Screening common DEGs in colorectal cancer by integrated microarray.

Common DEGs	Gene names
Upregulated	CXCL1, CXCL3, CXCL8, DUSP14, SULF1, SOX9, TGFBI, CDC25B, FAP, COL4A1, MMP7, HILPDA, VSNL1, KRT23, MMP1, S100A2, ANXA3, UBE2C, DDIT4
Downregulated	SPIB, SST, SLC4A4, CA7, AQP8, SCNN1B, GUCA2A, PYY, CA1, GCG, HPGD, HSD11B2, DHRS11, CA4, EDN3, ADAMDEC1, ZG16, HSD17B2, SLC26A3, CDHR5, CHGA, CLDN8, CA2, ABCA8, ABCG2, PTPRH, LRRC19, LGALS2, STMN2, TSPAN7, MS4A12, CDHR2, MXI1, SEPP1, CHP2, AKR1B10, ADH1B, AHCYL2, SELENBP1, TNFRSF17, SLC30A10, ENTPD5, CLCA4, CWH43, KRT20, SLC26A2, CEACAM7, BTNL8, SULT1A2, SLC25A20, ADH1C, NR3C2, DHRS9, DEFB1, CFD, CKB, CD177, ITM2A, GHR, TUBAL3, FABP4, DNASE1L3, RCAN2, C7, FHL1, PPP1R14D, EPB41L3, ADTRP

## Data Availability

GSE15781, GSE110223, and GSE110224 can be downloaded from the GEO database. URL of the GEO database: https://www.ncbi.nlm.nih.gov/geo/.

## Supplementary Materials

Supplementary 1 Table S1: detailed information regarding qRT-PCR primer of 10 core genes.

Supplementary 2 Table S2: GO analysis for upregulated common DEGs.

Supplementary 3 Table S3: GO analysis for downregulated common DEGs.
